# Mediating Effect of Physical Activity in the Association between Low 25-Hydroxyvitamin D and Frailty Trajectories: The English Longitudinal Study of Ageing

**DOI:** 10.3390/nu14112292

**Published:** 2022-05-30

**Authors:** Zaixing Shi, Kanglin Shi, Zeyun Zhang, Jianlin Lin, Ya Fang

**Affiliations:** 1 School of Public Health, Xiamen University, Xiamen 361000, China; zshi@xmu.edu.cn (Z.S.); 32620201150782@stu.xmu.edu.cn (K.S.); 32620201150769@stu.xmu.edu.cn (Z.Z.); jianlinlin@stu.xmu.edu.cn (J.L.); 2 State Key Laboratory of Molecular Vaccine and Molecular Diagnostics, School of Public Health, Xiamen University, Xiamen 361000, China; 3 Key Laboratory of Health Technology Assessment of Fujian Province, School of Public Health, Xiamen University, Xiamen 361000, China

**Keywords:** vitamin D, physical activity, frailty trajectories, mediation analysis

## Abstract

BACKGROUND: Frailty is associated with adverse health outcomes, and vitamin D (VD) deficiency may be a risk factor. We aimed to identify frailty trajectories and examine the mediating effect of physical activity (PA) on the association between VD deficiency and frailty trajectories. METHODS: We included 2997 participants aged 60 to 85 years from ELSA. VD was measured using serum 25-hydroxyvitamin D [25(OH)D] (sufficient: >50; insufficient: 30–50; deficient: <30 nmol/L). Frailty was assessed by a 60-item frailty index, and PA was measured on the basis of total energy expenditure. Frailty trajectories were identified using group-based trajectory modeling, and the mediation effect of PA was tested using causal mediation analysis. RESULTS: Three distinct frailty trajectories emerged: “Non-frail” (66.48%), “Pre-frail to frail” (25.67%) and “Frail to severely frail” (7.85%). VD deficiency was associated with the “Pre-frail to frail” (OR = 1.51, 95% CI: 1.14, 1.98) and “Frail to severely frail” trajectories (OR = 2.29, 95% CI: 1.45, 3.62). PA only mediated 48.4% (95% CI: 17.1%–270.8%) of the association between VD deficiency and the “Pre-frail to frail” trajectory. CONCLUSIONS: Vitamin D deficiency is associated with the onset and worsening of frailty in older adults, and reduced PA may mediate its impact on the transition from pre-frailty to frailty.

## 1. Introduction

Frailty is a clinical state characterized by reduced physiological function and reduced stress resistance in older adults, leading to adverse events including hospitalization, falls and death [1]. A recent systematic review of 240 studies from 62 countries suggested that the prevalence of pre-frailty and frailty measured by FI were 24% and 49% among individuals aged ≥50 years, respectively [2]. Multiple risk factors are associated with frailty, including older age, female sex, lower socioeconomic status, lower physical activity (PA) and poorer diet [3]. Of these, vitamin D (VD) is a potentially reversible risk factor because of its benefits to bone health and muscle strength. 25-Hydroxyvitamin D [25(OH)D] is the biologically active form of VD and a biomarker of VD status. 25(OH)D regulates calcium and phosphorus metabolism and maintains the balance of blood calcium and phosphorus [4]. VD deficiency [25(OH)D < 30 nmol/L] can increase the incidence of injuries and chronic diseases (e.g., cardiovascular disease) [5]. The prevalence of VD deficiency is high in older adults, with a quarter of adults aged 60 years and above having VD deficiency in the UK [6].

Previous studies on VD deficiency and frailty have predominantly focused on the binary frailty status. For instance, a cross-sectional study among individuals aged ≥65 years showed that those with 25(OH)D < 50 nmol/L had a 130% increased risk of frailty [7]. As a complex condition, frailty can be measured by nearly 70 instruments, of which the frailty phenotype (FP) and the frailty index (FI) are the most widely used [8]. FP comprises five domains: shrinking, weakness, poor endurance and energy, slowness and low physical activity level [9]; FI is based on accumulative health deficits, including restricted activity, disability, cognitive impairments, etc. [10]. Although the binary frailty status provides a convenient interpretation, it is not suitable for understanding the impact of VD deficiency on the gradual change in frailty status, as measured by the continuous FI score. Furthermore, frailty is a dynamically changing status, yet few have examined the type of developmental trajectories of frailty with age. Furthermore, the exact mechanisms underlying the association between VD deficiency and frailty remain unclear. Previous research suggested that VD deficiency may be correlated with physical inactivity, both of which may compromise the preservation of skeletal muscle and physical functioning and contribute to the development of frailty [11,12]. However, no study has prospectively evaluated whether PA mediates the association between VD deficiency and the development of frailty.

Utilizing data collected from the English Longitudinal Study of Ageing (ELSA), the current study aimed to (1) identify distinct frailty trajectories over 8 years among adults aged from 60 to 85 years in the UK, (2) examine the association between VD deficiency and frailty trajectories and (3) test the hypothesis that VD deficiency bears an indirect effect on frailty trajectories through low physical activity.

## 2. Methods

### 2.1. Data Source

This analysis used data from the English Longitudinal Study of Ageing (ELSA). ELSA is a nationally representative longitudinal survey of people aged 50 and over in England. The first survey was conducted in 2002 with participants from the Health Survey for England (HSE) samples in 1998, 1999 and 2001. Participants in ELSA were followed up every two years with a computer-assisted personal interview plus a self-administered questionnaire. The detailed study design of ELSA has been reported elsewhere [13]. We used longitudinal data from wave 6 (2012–2013) to wave 9 (2018–2020), because wave 6 was the earliest wave in which 25(OH)D data were available. A total of 10,601 participants enrolled in wave 6 of ELSA. We included participants who were ≥60 years and ≤85 years of age at the time of wave 6. Additionally, we excluded participants who had complete data for less than 60% of the 60 component variables of the frailty index and excluded those with missing baseline 25(OH)D concentration data. Ultimately, we included 2997 participants (Supporting Information Figure S1). The ELSA study was approved by the London Multicentre Research Ethics Committee, and all participants provided written informed consent.

### 2.2. Measures

#### 2.2.1. Serum 25(OH)D Concentration

The serum 25(OH)D levels were determined by the DiaSorin Liaison immunoassay performed on a fasting blood sample taken at the Royal Victoria Infirmary (Newcastle upon Tyne, United Kingdom) in wave 6′s nurse visits. In this study, 25(OH)D concentration was classified into three categories with reference to the Institute of Medicine (IOM) criteria: sufficient (≥50 nmol/L), insufficient (≥30 and <50 nmol/L) and deficient (<30 nmol/L) [14].

#### 2.2.2. Physical Activity (PA)

PA was measured by asking participants how often they engaged in vigorous, moderate and light activities. Each question had four options: never, one to three times a month, once a week and more than once a week. Participants were given specific examples of different PAs; for example, home repairs and laundry fell under vigorous PA, car washing and dancing fell under moderate PA, and cycling and swimming fell under light PA [15]. We estimated the total energy spent on PA by multiplying the duration and intensity of each type of PA. We used the following metabolic equivalent of task (MET) value to estimate the energy expenditure: light activities = 2 METs, moderate activities = 4 METs, and vigorous activities = 6 METs [16,17]. One MET is equivalent to the amount of energy produced relative to body mass in the resting state [18].

#### 2.2.3. Frailty Index (FI)

We used the frailty index to quantify the level of frailty. Based on the methodology of Searle et al. [10], we used 60 indicator variables representing six domains of health, including mobility difficulties, disability (ADL and IADL), chronic conditions, psychological problems and cognitive impairment (Supporting Information Table S1) [19]. The FI was calculated for all participants with at least 60% complete data for the 60 component variables. Each variable was scored between 0 and 1. For example, a binary variable (e.g., walk 100 yards) was assigned a value of 1 if the participant had difficulty and 0 if otherwise. For a five-category variable (e.g., self-reported general health), a response of “poor” was coded as 1, “fair” was coded as 0.75, “good” was coded as 0.5, “very good” was coded as 0.25 and “excellent” was coded as 0. For continuous variables such as immediate or delayed word recall, we recoded their upper quintile as 0, fourth quintile as 0.25, third quintile as 0.5, second quintile as 0.75 and lower quintile as 1. The FI was calculated by first summing all components and dividing by the number of non-missing variables. The FI also ranged between 0 and 1, where a higher FI means greater frailty. In previous analyses, an FI < 0.20 was considered non-frail, 0.20 ≤ FI < 0.35 was pre-frail, 0.35 ≤ FI < 0.43 was frail and FI ≥ 0.43 was severely frail [20,21].

#### 2.2.4. Covariates

Socio-demographic, anthropometric and health behavior factors from wave 6 were included as potential confounders. Socio-demographic variables included sex, education (higher education with degree, higher education below degree level, higher secondary school, lower secondary school or no education), marital status (married, single or never married, divorced or separated, or widowed), employment (employed or unemployed) and annual income (in quintiles). Health behaviors included smoking (never smoker, past smoker or current smoker), alcohol intake (less than once/week, times/week or daily) and BMI (normal: 18.5 kg/m^2^ ≤ BMI < 25 kg/m^2^; overweight: 25 kg/m^2^ ≤ BMI < 30 kg/m^2^; obese: BMI ≥ 30 kg/m^2^). Since the blood 25(OH)D level is subject to seasonal influences, we further adjusted for season [22]. VD supplement use was determined based on a question asking whether participants took calcium pills or vitamin D. A response of “Yes” was considered as the use of VD supplements.

### 2.3. Statistical Analyses

We used group-based trajectory modeling (GBTM) to identify distinct frailty trajectories with age. GBTM clusters individuals with similar trajectories into latent trajectory groups [23]. In this analysis, we fitted and compared GBTM with 2–6 trajectory groups and different shapes (linear, quadratic and cubic). The optimal model was determined based on (1) the minimum Akaike’s information criterion (AIC) and Bayesian information criteria (BIC) values; (2) the average posterior probability that each individual belonged to a certain trajectory group should be greater than 70%; and (3) each trajectory group should have 5% or higher membership [22]. We fitted the GBTM using the PROC TRAJ package in SAS and accounted for dropout or death using the DROPOUT option [24].

We conducted multinomial logistic regression to examine the association among different 25(OH)D levels, PA in wave 8 and frailty trajectories. For each association analysis, 4 models were fitted consecutively: Model 1 was the unadjusted model; Model 2 adjusted for basic demographic factors, including sex, education, marital status, employment, wealth, smoking, alcohol intake and BMI; Model 3 further adjusted for VD supplement use and season; and Model 4 further adjusted for PA in wave 6.

**Figure 1:**
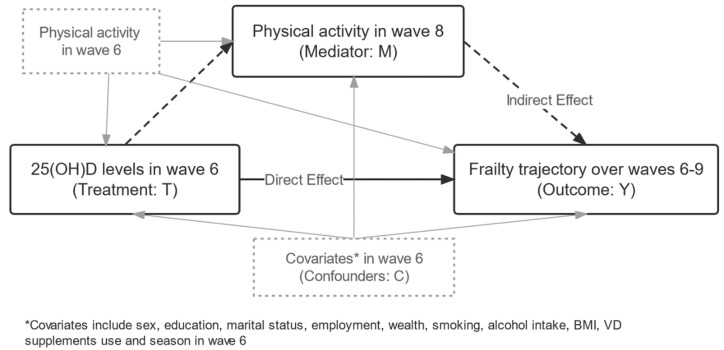
Directed acyclic graph of the current analysis. The exposure variable was 25(OH)D level in wave 6, the outcome variable was the latent frailty trajectory over waves 6–9, and the mediator variable was physical activity level in wave 8. The analysis adjusted for physical activity level in wave 6 and other potential confounders. The solid black lines represent a direct effect of 25(OH)D on frailty trajectories, and the dashed lines represent an indirect effect.

Causal mediation analysis was used to quantify the contribution of PA to the association between different 25(OH)D levels and frailty trajectories (Figure 1). Causal mediation analysis is an extension of the potential outcome framework [25], which is a non-parametric approach for evaluating the mediating effects of an individual mediator based on the assumption of sequential ignorability that the mediator could be independent of all potential outcomes conditional on the assigned treatment [26,27]. In the current study, let i=1,2,…,N denote the ith participant. The mediation model was specified as follows [26]:

$${\bar{\delta }}_{i}\left(t\right)\equiv \;E\left(Y_{i}\left(t,M_{i}\left(1\right)\right)-Y_{i}\left(t,M_{i}\left(0\right)\right)\right)$$

$${\bar{\zeta }}_{i}\left(t\right)\equiv \;E\left(Y_{i}\left(1,M_{i}\left(t\right)\right)-Y_{i}\left(0,M_{i}\left(t\right)\right)\right)$$

$$\bar{\tau }\equiv \;E\left(Y_{i}\left(1,M_{i}\left(1\right)\right)-Y_{i}\left(0,M_{i}\left(0\right)\right)\right)=\frac{1}{2}\overset{1}{\underset{t=0}{\sum }}\left\{{\bar{\delta }}_{i}\left(t\right)+{\bar{\zeta }}_{i}\left(t\right)\right\}$$

$M_{i}\left(T_{i}\right)$ denotes the value of the total energy spent on PA when individual i is observed to have a 25(OH)D concentration of $T_{i}$, which has two potential values of 0 and 1. $Y_{i}\left(T_{i},M_{i}\left(T_{i}\right)\right)$ denotes the potential frailty trajectory that would result if individual i has a 25(OH)D concentration of $T_{i}$ and a PA value of $M_{i}\left(T_{i}\right)$. Therefore, ${\bar{\delta }}_{i}\left(t\right)$ represents the average causal mediation effect (ACME), meaning the effect of 25(OH)D concentration on frailty trajectories through PA. ${\bar{\zeta }}_{i}\left(t\right)$ represents the average direct effect (ADE) of 25(OH)D concentration on frailty trajectories while controlling for the PA value. $\bar{\tau }$ represents the total effect (TE), which is the sum of ACME and ADE. We defined participants with sufficient VD as the control group and those with insufficient and deficient VD as the treatment groups. The causal mediation analyses adjusted for sex, education, marital status, employment, wealth, smoking, alcohol intake, BMI, VD supplement use, season and PA in wave 6. We also conducted subgroup analysis to evaluate the mediation effect of PA in different populations at high risk of frailty, including participants with falls, depression, loneliness, living alone, hypertension, diabetes, arthritis, obesity, smoking and social isolation.

Data cleaning and analysis were conducted in R version 4.0.4 (R Core Team, Vienna, Austria). GBTM was performed in SAS (version 9.4, SAS Institute, Cary, NC, USA) using PROC TRAJ. Mediation analysis was performed using the R package “mediation”, and *p* <0.05 was regarded as statistically significant.

## 3. Results

### 3.1. Sample Characteristics

The sample included 2997 individuals with an average age of 68.6 years. Most participants were female (55.5%), were married (68.9%), were unemployed (79.5%), had no formal education (37.5%), were never smokers (89.5%), were overweight (45.1%), drank a few times per week (45.2%) and engaged in moderate PA (49.5%). The prevalence of VD insufficiency and deficiency was 32.1% and 19.6%, respectively (Table 1).

### 3.2. Frailty Trajectories

**Figure 2:**
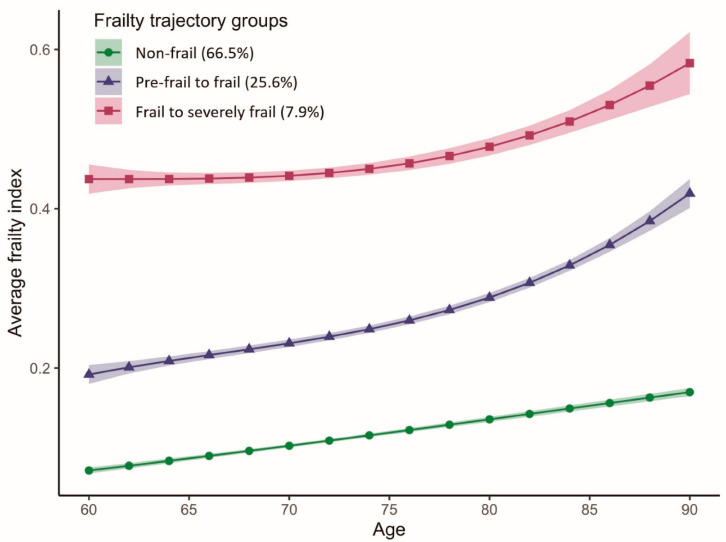
Average frailty index with age according to frailty trajectories among older adults in the ELSA study. The solid lines represent the means, and the ribbons represent the 95% confidence intervals of the mean.

We identified three latent frailty trajectories for individuals who were 60 to 85 years of age (Figure 2). The first group (n = 2837, 66.48%) maintained an average FI below 0.20 and thus was labeled “Non-frail.” The average FI for the second group (n = 1067, 25.67%) increased rapidly from 0.19 to 0.42 (FI > 0.35), and therefore, the group was labeled “Pre-frail to frail”. The third group (n = 333, 7.85%) showed an increasing FI from 0.44 to 0.58, meaning that the participants transitioned from being frail to severely frail and were thus labeled “Frail to severely frail”. Participants in the “Frail to severely frail” group were more likely to have VD deficiency, be female, married and unemployed, have a lower level of education and wealth, have never smoked, be obese, drink less than once a week, not use VD supplements and report no PA, compared to those in the “Non-frail” group (Table 1).

### 3.3. Association between 25(OH)D Levels and Frailty Trajectories

In unadjusted multivariable logistic regression, participants with insufficient serum VD levels were more likely to follow the “Pre-frail to frail” trajectory and the “Frail to severely frail” trajectory (OR = 1.32, 95% CI: 1.09–1.61; OR = 1.55, 95% CI: 1.09–2.20, respectively, Table 2) compared to those with sufficient VD. However, this association disappeared when adjusting for confounders. Older adults with deficient VD were more likely to be in the “Pre-frail to frail” trajectory and “Frail to severely frail” trajectory (OR = 1.51, 95% CI: 1.14–1.98; OR = 2.29, 95% CI: 1.45–3.62, respectively) compared to those with sufficient VD. VD deficiency was significantly associated with the “Frail to severely frail” trajectory, even after controlling for all confounders (OR = 1.79, 95% CI: 1.11–2.88).

### 3.4. Causal Mediation Analysis of the Mediating Effect of Physical Activity

In the causal mediation analysis of the risk of being in the “Pre-frail to frail” trajectory group versus the “Non-frail” group, PA showed a significant ACME on VD deficiency (OR = 1.024, 95% CI: 1.014–1.036), which mediated 48.4% (95% CI: 17.1%–270.8%; *p* = 0.040) of the association. However, we did not observe any significant ACME on the association between VD insufficiency and the “Pre-frail to frail” trajectory. In the causal mediation analysis of the risk of being in the “Frail to severely frail” trajectory group versus the “Non-frail” group, PA did not show a significant ACME.

**Figure 3:**
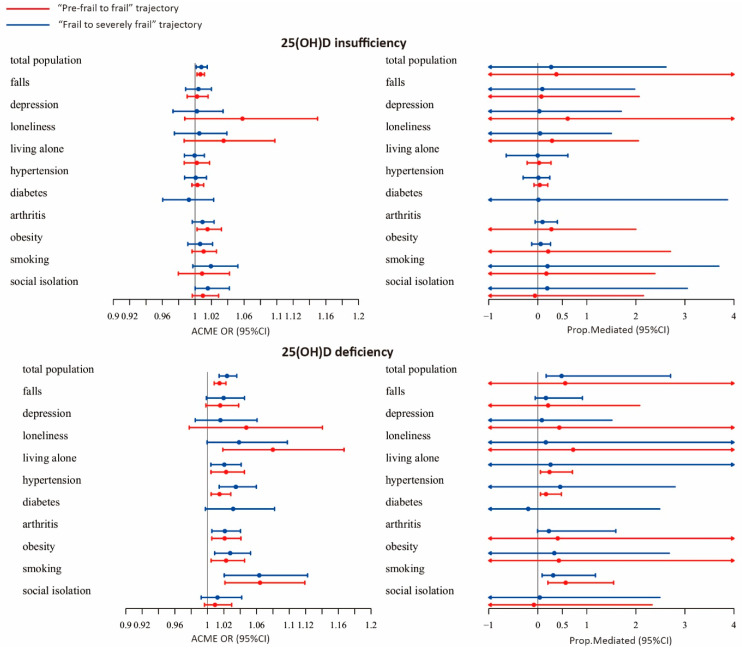
Forest plot of the average causal mediation effect (ACME) of physical activity.

In the subgroup analysis, PA showed a significant mediation effect on the association between VD deficiency and the “Pre-frail to frail” trajectory among older adults who smoked (ACME = 1.063, 95% CI: 1.02–1.12, proportion mediated = 31.5%). In addition, PA showed a significant mediation effect on the association of VD deficiency with the “Frail to severely frail” trajectory among older adults who lived alone (ACME = 1.02, 95% CI: 1.004–1.05, proportion mediated = 23.9%), had hypertension (ACME = 1.02, 95% CI: 1.004–1.03, proportion mediated = 16.8%) and smoked (ACME = 1.064, 95% CI: 1.02–1.12, proportion mediated = 56.7%) (Figure 3; Supporting Information Table S5).

## 4. Discussion

Based on a large cohort of older adults in the UK, our analyses identified three distinct types of frailty trajectories over the ages of 60 to 85 years. Our results suggested that participants with VD deficiency had a greater likelihood of following the “Pre-frail to frail” and the “Frail to severely frail” trajectories relative to the “Non-frail” trajectory. In the causal mediation analysis, PA mediated approximately 48.4% of the association between VD deficiency and the “Pre-frail to frail” trajectory, but not the “Frail to severely frail” trajectory. These findings suggest that VD deficiency may accelerate the transition from pre-frailty to frailty through reduced physical activity. Therefore, it is important to promote physical activity for the primary prevention of frailty among older adults suffering from VD deficiency.

Limited studies have explored heterogeneous developmental frailty trajectories. Our study identified three distinct types of frailty trajectories: “Non-frail”, “Pre-frail to frail” and “Frail to severely frail”. Similar to our findings, Howrey et al. identified three groups of frailty: “non-frail”, “moderate progressive frailty” and “progressive high frailty” among older Mexican Americans [28]. However, this study used the survey wave as the underlying time scale, which is not suitable for analyzing the trajectory of age-related phenotypes. Recently, Mandelblatt et al. measured frailty with a 42-item deficit accumulation index and revealed three frailty trajectories using a growth mixture model among older adults with breast cancer and non-cancer controls: “remain robust”, “remain frail” and “became frailer” [29]. However, these results may be biased by their sample of cancer survivors and have limited generalizability to the general population. A prospective study from Spain that included 975 older adults identified five groups of frailty trajectories: “non-frailty”, “improving to non-frailty”, “developing frailty”, “remaining frail” and “increasing frailty” [30]. The study used a novel frailty measurement instrument, the Frailty Trait Scale (FTS5), which is not widely applied, and its validity has not been evaluated. Furthermore, they included only two frailty measurements five years apart; thus, the results were unable to reflect the continuous change in frailty over the longer term.

Although previous studies have investigated whether VD is associated with frailty, few have examined its association with the developmental trajectories of frailty. A recent systematic review [31] of 26 studies unequivocally suggested that a lower 25(OH)D concentration is associated with a higher risk of frailty. The present study further demonstrates that older adults with VD deficiency may be at greater risk of the onset and worsening of frailty. Although the exact mechanism remains unclear, VD may exert its effects on frailty mainly through its regulation of bones and muscles. VD deficiency affects the intestinal absorption of calcium, resulting in less calcium for bone mineralization, and may increase parathyroid hormone, which stimulates bone metabolism and accelerates bone loss [32]. In addition, individuals with deficient VD have reduced muscle fibers, particularly type II muscle fibers, which play important roles in moderate to vigorous activities [33]. The loss of body mass significantly undermines one’s ability to perform physical exercises, which causes further losses of physical functions. Therefore, PA may mediate the impact of VD deficiency on frailty. Our data support the hypothesis that PA mediates the association between VD deficiency and the frailty trajectory. This suggests that PA is a potential protective factor against frailty for older adults with deficient VD.

Our study has several advantages over previous studies. First, we gridded the longitudinal frailty assessment data by age; that is, we used age as the underlying time scale, which allowed us to better understand how frailty changes with age in older adults. Second, we took baseline PA into account to minimize reverse causation. However, our study has several limitations. First, we only examined VD at baseline, which ignores changes in VD over time. Second, some components of the FI were long-term or irreversible, which made it difficult to observe any improvement in frailty. Finally, most variables of the FI were self-reported, which makes them subject to recall bias and may lead to inaccurate estimates of the frailty trajectories.

## 5. Conclusions

Based on a nationally representative sample of older adults in the UK, we identified three distinct frailty trajectories: “Non-frail”, “Pre-frail to frail” and “Frail to severely frail.” Older adults with insufficient and deficient VD were at significantly higher risk of following the “Pre-frail to frail” and the “Frail to severely frail” trajectories. PA mediated approximately half of the association between VD deficiency and the “Pre-frail to frail” trajectory. Our results highlight the importance of developing strategies to promote physical activities to prevent the onset of frailty in older adults with low levels of VD. Given the observational nature of this analysis, interventional studies are needed to investigate the protective effect of PA in reducing frailty among older adults with VD deficiency.

## Figures and Tables

**Table 1:** Baseline characteristics by frailty trajectory.

Variables	Total (n = 2997)	Non-Frail (n = 2059)	Pre-Frail to Frail (n = 724)	Frail to Severely Frail (n = 214)	*p*-Value *
**Frailty index, mean (SD)**	0.15 (0.11)	0.09 (0.05)	0.23 (0.08)	0.42 (0.12)	<0.001
**Age (years), mean (SD)**	68.6 (6.3)	68.3 (6.1)	69.7 (6.8)	68.4 (6.3)	0.001
**Sex, n (%)**					<0.001
Male	1334 (44.5)	1030 (50.0)	244 (33.7)	60 (28.0)	
Female	1663 (55.5)	1029 (50.0)	480 (66.3)	154 (72.0)	
**Education, n (%)**					<0.001
Degree	558 (18.7)	463 (22.6)	80 (11.1)	15 (7.0)	
Higher education	469 (15.7)	347 (17.0)	96 (13.3)	26 (12.2)	
Higher secondary	264 (8.9)	183 (8.9)	70 (9.7)	11 (5.2)	
Lower secondary	572 (19.2)	391 (19.1)	141 (19.5)	40 (18.8)	
No formal qualifications	1119 (37.5)	662 (32.4)	336 (46.5)	121 (56.8)	
**Marital status, n (%)**					<0.001
Married	2065 (68.9)	1509 (73.4)	444 (61.3)	112 (52.3)	
Single or never married	145 (4.8)	105 (5.1)	29 (4.0)	11 (5.1)	
Divorced or separated	361 (12.1)	214 (10.4)	99 (13.7)	48 (22.4)	
Widowed	424 (14.2)	229 (11.1)	152 (21.0)	43 (20.1)	
**Employment, n (%)**					<0.001
Employed	609 (20.5)	502 (24.6)	99 (13.8)	8 (3.8)	
Unemployed	2358 (79.5)	1535 (75.4)	619 (86.2)	204 (96.2)	
**Annual income, n (%)**					<0.001
Fifth quintile (≥£141,500)	614 (20.8)	501 (24.7)	98 (13.6)	15 (7.1)	
Fourth quintile (≥£59,000 and <£141,500)	598 (20.2)	474 (23.3)	113 (15.7)	11 (5.2)	
Third quintile (≥£23,720 and <£59,000)	595 (20.1)	436 (21.5)	130 (18.1)	29 (13.8)	
Second quintile (≥£5500 and <£23,720)	588 (19.9)	355 (17.5)	182 (25.3)	51 (24.3)	
Lower quintile (<£5500)	564 (19.1)	264 (13.0)	196 (27.3)	104 (49.5)	
**Smoking, n (%)**					<0.001
Never smoker	2681 (89.5)	1871 (90.9)	634 (87.6)	176 (82.2)	
Past smoker	71 (2.4)	53 (2.6)	16 (2.2)	2 (0.9)	
Current smoker	245 (8.2)	135 (6.6)	74 (10.2)	36 (16.8)	
**Alcohol intake, n (%)**					<0.001
Less than once a week	1087 (38.3)	615 (31.3)	350 (51.2)	122 (63.2)	
Once to six days a week	1284 (45.2)	987 (50.3)	252 (36.9)	45 (23.3)	
Daily	467 (16.5)	360 (18.3)	81 (11.9)	26 (13.5)	
**BMI, n (%)**					<0.001
Normal	728 (24.9)	586 (29.0)	117 (16.6)	25 (12.7)	
Pre-obese	1317 (45.1)	966 (47.8)	291 (41.2)	60 (30.5)	
Obese	878 (30.0)	468 (23.2)	298 (42.2)	112 (56.9)	
**Vitamin D supplements use, n (%)**					<0.001
No	2850 (95.1)	1986 (96.5)	673 (93.0)	191 (89.3)	
Yes	147 (4.9)	73 (3.5)	51 (7.0)	23 (10.7)	
**25(OH)D level, n (%)**					<0.001
≥50 nmol/L (Sufficient)	1448 (48.3)	1077 (52.3)	301 (41.6)	70 (32.7)	
≥30 and <50 nmol/L (Insufficient)	963 (32.1)	655 (31.8)	242 (33.4)	66 (30.8)	
<30 nmol/L (Deficient)	586 (19.6)	327 (15.9)	181 (25.0)	78 (36.4)	
**Physical Activity, n (%)**					<0.001
Vigorous	1032 (34.4)	874 (42.4)	139 (19.2)	19 (8.9)	
Moderate	1485 (49.5)	1015 (49.3)	400 (55.2)	70 (32.7)	
None	480 (16.0)	170 (8.3)	185 (25.6)	125 (58.4)	

* *p*-values were obtained from Chi-squared test.

**Table 2:** Association between serum 25(OH)D level and frailty trajectories in all participants and in subgroups with specific risk factors.

Group	25(OH)D Level (Ref: ≥50 nmol/L)	Non-Frail (Ref)	Model 1		Model 2		Model 3		Model 4	
			Pre-Frail to Frail	Frail to Severely Frail	Pre-Frail to Frail	Frail to Severely Frail	Pre-Frail to Frail	Frail to Severely Frail	Pre-Frail to Frail	Frail to Severely Frail
			OR (95%CI)	OR (95%CI)	OR (95%CI)	OR (95%CI)	OR (95%CI)	OR (95%CI)	OR (95%CI)	OR (95%CI)
**Total population** **(n = 2997)**	30–50		1.32 ** (1.09, 1.61)	1.55 * (1.09, 2.2)	1.15 (0.93, 1.44)	1.13 (0.74, 1.72)	1.24 (0.99, 1.55)	1.29 (0.84, 1.99)	1.15 (0.91, 1.44)	1.23 (0.79, 1.92)
	<30		1.98 ** (1.59, 2.47)	3.67 ** (2.6, 5.18)	1.37 * (1.06, 1.78)	1.98 ** (1.29, 3.02)	1.51 ** (1.14, 1.98)	2.29 ** (1.45, 3.62)	1.29 (0.97, 1.71)	1.79 * (1.11, 2.88)
**Fall** **(n = 789)**	30–50		1.14 (0.8, 1.63)	1.43 (0.85, 2.43)	1.08 (0.72, 1.63)	1.22 (0.63, 2.38)	1.15 (0.76, 1.75)	1.36 (0.69, 2.7)	1.12 (0.73, 1.72)	1.57 (0.77, 3.22)
	<30		2.36 ** (1.54, 3.61)	4.30 ** (2.48, 7.43)	1.87 * (1.13, 3.11)	2.70 ** (1.29, 5.67)	1.99 * (1.16, 3.42)	3.11 ** (1.40, 6.92)	1.75( 1.00, 3.05)	2.35 * (1.02, 5.45)
**Depression** **(n = 273)**	30–50		2.08 (0.98, 4.39)	2.30 * (1, 5.27)	1.72 (0.68, 4.30)	1.10 (0.38, 3.25)	1.85 (0.71, 4.87)	1.23 (0.39, 3.86)	1.38 (0.48, 3.93)	0.78 (0.23, 2.72)
	<30		1.96 (0.84, 4.56)	4.65 ** (1.95, 11.08)	1.45 (0.51, 4.16)	2.35 (0.75, 7.41)	2.32 (0.70, 7.75)	3.85 * (1.02, 14.54)	1.71 (0.48, 6.11)	2.02 (0.48, 8.45)
**Living alone** **(n = 760)**	30–50		1.44 * (1, 2.07)	2.23 * (1.18, 4.21)	1.27 (0.83, 1.93)	1.91 (0.89, 4.09)	1.41 (0.92, 2.17)	2.68 * (1.18, 6.06)	1.33 (0.85, 2.07)	3.06 *(1.29, 7.26)
	<30		1.32 (0.88, 1.98)	4.08 ** (2.21, 7.54)	1.12 (0.70, 1.8)	3.48 ** (1.67, 7.24)	1.31 (0.79, 2.19)	4.87 ** (2.11, 11.24)	1.04 (0.61, 1.77)	3.78 ** (1.57, 9.11)
**Hypertension** **(n = 1030)**	30–50		1.60 ** (1.17, 2.19)	2.16 ** (1.26, 3.72)	1.41 (0.98, 2.02)	1.68 (0.84, 3.37)	1.56 * (1.08, 2.25)	1.93 (0.94, 3.97)	1.54 * (1.06, 2.24)	2.31 * (1.06, 5.01)
	<30		1.91 ** (1.34, 2.71)	4.91 ** (2.91, 8.28)	1.44 (0.96, 2.16)	3.36 ** (1.73, 6.53)	1.72 * (1.11, 2.67)	4.75 ** (2.28, 9.93)	1.47 (0.94, 2.32)	3.42 ** (1.54, 7.61)
**Diabetes** **(n = 227)**	30–50		1.21 (0.62, 2.36)	2.90 * (1.05, 8.05)	1.08 (0.47, 2.49)	3.03 (0.68, 13.48)	1.17 (0.5, 2.73)	5.00 (0.88, 28.46)	1.12 (0.45, 2.82)	10.02 * (1.01, 99.69)
	<30		1.32 (0.65, 2.66)	3.57 * (1.27, 10.04)	0.79 (0.32, 1.95)	3.20 (0.73, 14.06)	0.72 (0.27, 1.89)	5.98 * (1.02, 35.11)	0.36 (0.12, 1.10)	2.82 (0.32, 24.87)
**Arthritis** **(n = 1100)**	30–50		1.69 ** (1.25, 2.29)	2.03 ** (1.32, 3.13)	1.45 * (1.02, 2.06)	1.47 (0.88, 2.48)	1.59 * (1.11, 2.27)	1.67 (0.97, 2.85)	1.54 * (1.07, 2.22)	1.68 (0.96, 2.94)
	<30		2.23 ** (1.58, 3.14)	3.98 ** (2.56, 6.17)	1.46 (0.98, 2.2)	1.92 * (1.11, 3.32)	1.67 * (1.08, 2.56)	2.26 ** (1.26, 4.06)	1.47 (0.94, 2.29)	1.79 (0.97, 3.33)
**Obesity** **(n = 878)**	30–50		1.54 * (1.10, 2.17)	1.44 (0.86, 2.40)	1.68 ** (1.15, 2.45)	1.34 (0.74, 2.44)	1.70 ** (1.16, 2.50)	1.41 (0.77, 2.58)	1.63 * (1.09, 2.41)	1.46 (0.78, 2.75)
	<30		1.72 ** (1.18, 2.52)	2.53 ** (1.50, 4.26)	1.53 (0.99, 2.36)	2.02 * (1.09, 3.73)	1.60 * (1.02, 2.53)	2.26 * (1.18, 4.34)	1.35 (0.84, 2.17)	1.89 (0.95, 3.78)
**Smoking** **(n = 316)**	30–50		0.99 (0.53, 1.86)	1.22 (0.42, 3.55)	1.19 (0.56, 2.51)	1.18 (0.31, 4.54)	1.35 (0.62, 2.94)	1.27 (0.32, 5.00)	1.19 (0.53, 2.66)	1.31 (0.33, 5.27)
	<30		2.24 * (1.21, 4.17)	5.73 ** (2.27, 14.46)	2.89 ** (1.35, 6.19)	5.70 ** (1.76, 18.48)	3.95 ** (1.67, 9.36)	9.37 ** (2.60, 33.74)	2.99 * (1.22, 7.37)	7.26 ** (1.94, 27.17)
**Social isolation** **(n = 413)**	30–50		1.48 (0.88, 2.49)	0.80 (0.35, 1.82)	1.37 (0.74, 2.51)	0.51 (0.18, 1.43)	1.38 (0.74, 2.58)	0.55 (0.19, 1.63)	1.41 (0.74, 2.70)	0.57 (0.17, 1.91)
	<30		2.73 ** (1.52, 4.90)	2.40 * (1.10, 5.21)	1.40 (0.70, 2.79)	1.14 (0.44, 2.92)	1.39 (0.67, 2.91)	1.01 (0.34, 3.00)	1.03 (0.47, 2.23)	0.61 (0.19, 2.01)

Note: Abbreviations: OR, odds ratio; CI, confidence interval. Model 1 is the unadjusted model. Model 2 was further adjusted for sex, education, marital status, employment, wealth, smoking, alcohol intake and BMI. Model 3 was further adjusted for VD supplement use and season. Model 4 was further adjusted for physical activity in wave 6.

## Data Availability

Publicly available datasets were analyzed in this study. The ELSA study data can be downloaded from UK Data Service: https://beta.ukdataservice.ac.uk/datacatalogue/series/series?id=200011 access on 14 May 2022.

## Supplementary Materials

The following supporting information can be downloaded at: https://www.mdpi.com/article/10.3390/nu14112292/s1, Table S1. Prevalence of FI component variables in baseline (n = 4237), Table S2. Fit statistics for FI trajectories, Table S3. Maximum Likelihood Estimates of FI trajectories, Table S4. Linear regression between serum 25(OH)D level in wave 6 (treatment) and PA in wave 8 (mediator) in all participants and in subgroups with specific risk factors, Table S5. Causal mediation analysis of the mediating effect of physical activity, Table S6. Association between different 25(OH)D concentration and frailty trajectories by gender, Figure S1. The English Longitudinal Study of Ageing (ELSA) recruitment

## Abbreviations

ELSA: the English Longitudinal Study of Ageing; VD: vitamin D; PA: physical activity; 25(OH)D: 25-hydroxyvitamin D; FI: frailty index; BIC: Bayesian information criterion; AIC: Akaike’s information criterion.
