# Supplementary material for: Mediating Effect of Physical Activity in the Association between Low 25-Hydroxyvitamin D and Frailty Trajectories: The English Longitudinal Study of Ageing
Source: Nutrients. 2022 May 30;14(11):2292. doi: 10.3390/nu14112292 (PMC9183055; doi:10.3390/nu14112292)
Supplement: Supplementary file 1 [file nutrients-14-02292-s001.zip › nutrients-1749434-supplementary.pdf]

Supporting Information Figure 1. The English Longitudinal Study of Ageing (ELSA) recruitment

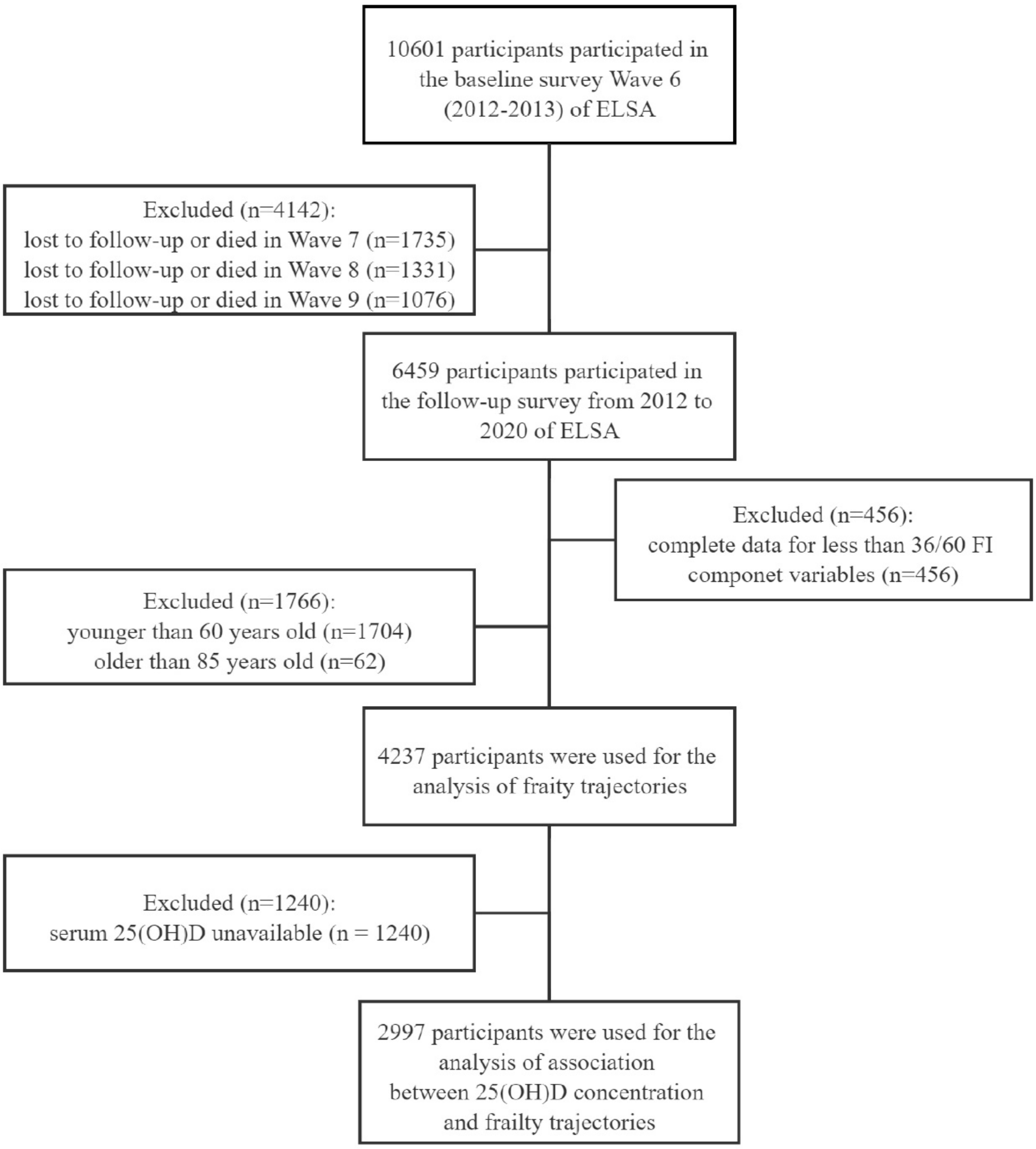

Supporting Information Table 1. Prevalence of FI component variables in baseline (N=4237)

Domain	FI components	N(%)
**Mobility Difficulties**	Having difficulty walking 100 yards	374 (8.8)
	Having difficulty sitting for about two hours	512 (12.1)
	Unable to get up from a chair after sitting for long periods	968 (22.8)
	Unable to climb several flights of stairs without resting	1272 (30.0)
	Unable to climb one flight of stairs without resting	437 (10.3)
	Having difficulty stooping, kneeling, or crouching	1503 (35.5)
	Unable to reach or extending arms above shoulder level	410 (9.7)
	Unable to pull or pushing large objects like a living room chair	556 (13.1)
	Unable to lift or carrying weights over 10 pounds, like a heavy bag	779 (18.4)
	Unable to pick up a 5p coin from a table	196 (4.6)
**Disability (ADL and IADL)**	Having difficulty dressing, including putting on shoes and socks	409 (9.7)
	Having difficulty walking across a room	73 (1.7)
	Having difficulty bathing or showering	250 (5.9)
	Having difficulty eating, such as cutting up your food	54 (1.3)
	Having difficulty getting in or out of bed	164 (3.9)
	Unable to use the toilet, including getting up or down	104 (2.5)
	Unable to use a map to figure out how to get around in a strange place	106 (2.5)
	Having difficulty preparing a hot meal	92 (2.2)
	Having difficulty shopping for groceries	189 (4.5)
	Having difficulty making telephone calls	44 (1.0)
	Having difficulty taking medications	22 (0.5)
	Having difficulty managing money	42 (1.0)
	Having difficulty doing work around the house or garden	449 (10.6)
**Psychological Problems**	Respondent felt depressed much of the time during past week	421 (9.9)
	Respondent felt everything they did during the past week was an effort	598 (14.1)
	Respondent felt their sleep was restless during the past week	1357 (32.0)
	Respondent was not happy much of the time during the past week	349 (8.2)
	Respondent felt lonely much of the time during the past week	395 (9.3)
	Respondent didn't enjoy life much of the time during the past week	323 (7.6)
	Respondent felt sad much of the time during the past week	685 (16.2)
	Respondent could not get going much of the time during the past week	651 (15.4)
**Chronic Conditions**	Confirming high blood pressure or hypertension	1534 (36.2)
	Confirming angina	204 (4.8)
	Confirming heart attack	173 (4.1)

	Confirming congestive heart failure	14 (0.3)
	Confirming an abnormal heart rhythm	252 (5.9)
	Confirming diabetes or high blood sugar	380 (9.0)
	Confirming a stroke (cerebral vascular disease)	118 (2.8)
	Confirming chronic lung disease such as chronic bronchitis or emphysema	126 (3.0)
	Confirming asthma	412 (9.7)
	Confirming arthritis (including osteoarthritis , or rheumatism)	1540 (36.3)
	Confirming osteoporosis, sometimes called thin or brittle bones	256 (6.0)
	Confirming cancer or a malignant tumour (excluding minor skin cancers)	128 (3.0)
	Confirming parkinson's disease	11 (0.3)
	Confirming any emotional, nervous or psychiatric problems	342 (8.1)
	Confirming alzheimer's disease	0 (0.0)
	Confirming dementia, or any other serious memory impairment	8 (0.2)
**Other Self-reported Conditions**	Self-reported general health(fair or poor)	934 (22.0)
	Self-reported eyesight(fair, poor or blind)	434 (10.2)
	Self-reported hearing(fair or poor)	887 (20.9)
	Respondent had fallen down	1125 (26.6)
	Respondent had fractured hip	16 (0.4)
	Respondent had joint replacement	158 (3.7)
	Respondent had pain whilst walking	400 (9.4)
**Cognitive Impairment**	Identify today's date incorrectly: day of month	562 (13.3)
	Identify today's date incorrectly: month	59 (1.4)
	Identify today's date incorrectly: year	30 (0.7)
	Identify the day of the week incorrectly	31 (0.7)
	Immediate word recall(less than 6 words)	1336 (31.5)
	Delayed word recall(less than 4 words)	778 (18.4)

Supporting Information Table 2. Fit statistics for FI trajectories

Fit statistic	Number of classes				
	1	2	3	4	5
**BIC**	11402.63	16475.86	18405.88	19186.52	19516.21
**AIC**	11421.68	16513.97	18466.22	19250.03	19595.61
**Class proportion**	Class1,100%	Class1, 82.27%	Class1,66.48%	Class1,26.51%	Class1,2.48%
		Class2,17.73%	Class2,25.67%	Class2,61.06%	Class2,25.53%
			Class3,7.85%	Class3,9.49%	Class3,50.71%
				Class4,2.93%	Class4,14.62%
					Class5,6.67%
**AvePP**		Class1,0.99	Class1,0.97	Class1,0.91	Class1,0.97
		Class2,0.96	Class2,0.94	Class2,0.96	Class2,0.82
			Class3,0.97	Class3,0.94	Class3,0.92
				Class4,0.98	Class4,0.88
					Class5,0.95

Supporting Information Table 3. Maximum Likelihood Estimates of FI trajectories

Group	Parameter	Estimate	SE	Z value	p value
**Class 1: Non-frail (N=2837, 66.48%)**	Intercept	-0.142	0.008	-17.488	<.001
	Linear	0.003	<.001	30.175	<.001
**Class 2: Pre-frail to frail (N=1067, 25.67%)**	Intercept	-2.735	1.100	-2.485	0.013
	Linear	0.126	0.045	2.789	0.005
	Quadratic	-0.002	0.001	-2.993	0.003
	Cubic	<.001	<.001	3.323	0.001
**Class 3: Frail to severely frail (N=333, 7.85%)**	Intercept	-0.942	2.010	-0.469	0.639
	Linear	0.068	0.083	0.817	0.414
	Quadratic	-0.001	0.001	-0.977	0.329
	Cubic	<.001	<.001	1.174	0.240

Supporting Information Table 4. Linear regression between serum 25(OH)D level in wave 6 (treatment) and PA in wave 8 (mediator) in all participants and in subgroups with specific risk factors

Group	25(OH)D level (ref=≥50 nmol/L)	Non-frail	Model 1<br>OR (95%CI)	Model 2<br>OR (95%CI)	Model 3<br>OR (95%CI)	Model 4<br>OR (95%CI)
**Total population (N=2,997)**	30-50		-2.13** (-3.07,-1.20)	-1.55** (-2.48,-0.63)	-1.86** (-2.8,-0.92)	-1.00* (-1.87,-0.13)
	<30		-6.44** (-7.54,-5.34)	-4.28** (-5.41,-3.14)	-4.77** (-5.96,-3.59)	-2.95** (-4.05,-1.84)
**Fall (N=789)**	30-50		-0.84 (-2.76,1.07)	-0.36 (-2.22,1.50)	-0.59 (-2.48,1.30)	-0.07 (-1.83,1.68)
	<30		-6.26** (-8.52,-4.00)	-4.03** (-6.36,-1.69)	-4.50** (-6.97,-2.03)	-2.80* (-5.10,-0.50)
**Dpression (N=273)**	30-50		-3.24* (-6.37,-0.11)	-2.86 (-6.18,0.47)	-2.82 (-6.16,0.53)	-1.27 (-4.60,2.07)
	<30		-5.87** (-9.08,-2.66)	-4.27* (-7.79,-0.75)	-4.65* (-8.35,-0.96)	-2.55 (-6.21,1.11)
**Loneliness (N=264)**	30-50		-1.66 (-4.78,1.47)	-0.43 (-3.93,3.07)	-1.52 (-5.12,2.08)	-0.47 (-3.79,2.85)
	<30		-6.90** (-10.16,-3.65)	-6.00** (-9.73,-2.26)	-7.19** (-11.11,-3.28)	-4.43* (-8.10,-0.75)
**Living alone (N=760)**	30-50		-0.96 (-2.89,0.97)	-0.24 (-2.14,1.66)	-0.84 (-2.75,1.07)	-0.13 (-1.89,1.63)
	<30		-5.35** (-7.43,-3.27)	-3.82** (-5.92,-1.73)	-5.01** (-7.23,-2.78)	-2.93** (-5.00,-0.86)
**Hypertension (N=1030)**	30-50		-1.05 (-2.67,0.56)	-0.19 (-1.78,1.4)	-0.51 (-2.11,1.08)	-0.23 (-1.71,1.25)
	<30		-6.93** (-8.71,-5.15)	-5.24** (-7.02,-3.45)	-6.02** (-7.91,-4.13)	-3.95** (-5.73,-2.16)
**Diabetes (N=227)**	30-50		-1.82 (-5.28,1.65)	-0.54 (-3.97,2.88)	-0.47 (-3.97,3.03)	0.43 (-2.91,3.77)
	<30		-7.37** (-10.97,-3.77)	-6.29** (-9.90,-2.68)	-5.80** (-9.63,-1.97)	-3.88* (-7.62,-0.15)
**Arthritis (N=1100)**	30-50		-2.39** (-3.95,-0.83)	-1.23 (-2.78,0.33)	-1.54 (-3.12,0.03)	-1.08 (-2.54,0.37)
	<30		-6.06** (-7.76,-4.37)	-3.50** (-5.27,-1.73)	-4.05** (-5.90,-2.20)	-2.52** (-4.24,-0.80)
**Obese (N=878)**	30-50		-1.45 (-3.16,0.27)	-1.18 (-2.9,0.55)	-1.17 (-2.91,0.56)	-0.56 (-2.17,1.05)
	<30		-5.01** (-6.90,-3.12)	-4.38** (-6.33,-2.42)	-4.30** (-6.35,-2.26)	-2.56** (-4.48,-0.65)
**Smoking (N=316)**	30-50		-1.53 (-4.55,1.48)	-2.91 (-5.84,0.02)	-3.04* (-5.97,-0.11)	-1.90 (-4.69,0.90)
	<30		-9.05** (-12.08,-6.01)	-8.48** (-11.54,-5.43)	-9.53** (-12.78,-6.28)	-7.03** (-10.19,-3.88)
**Social isolation (N=413)**	30-50		-2.41 (-4.94,0.12)	-1.78 (-4.37,0.81)	-2.14 (-4.78,0.50)	-2.35* (-4.60,-0.11)
	<30		-7.87** (-10.77,-4.97)	-4.55** (-7.61,-1.50)	-5.30** (-8.53,-2.07)	-2.13 (-4.92,0.65)

Note: Abbreviations: OR, odds ratio; CI, confidence interval.Model 1 was the unadjusted model. Model 2 was further adjusted for sex, education, marital status, employment, wealth, smoking, alcohol intake and BMI. Model 3 was further adjusted for VD supplements use and season. Model 4 was further adjusted for physical activity at wave 6. *P < 0.05; **P < 0.01

Supporting Information Table 5. Causal mediation analysis of the mediating effect of physical activity

Group	25(OH)D level (ref: ≥50 nmol/L)	Parameter	Non-frail	Pre-frail to frail		Frail to severely frail	
				Estimate	95%CI	Estimate	95%CI
Total population (N=2,997)	30-50	ACME		1.008*	(1.001,1.015)	1.007**	(1.002,1.011)
		ADE		1.018	(0.982,1.055)	1.007	(0.984,1.032)
		TE		1.026	(0.989,1.066)	1.014	(0.99,1.039)
		Prop. Mediated		0.272	(-1.037,2.615)	0.377	(-4.092,5.587)
	<30	ACME		1.024**	(1.014,1.036)	1.015**	(1.008,1.022)
		ADE		1.025	(0.979,1.073)	1.011	(0.983,1.04)
		TE		1.049*	(1.002,1.099)	1.026	(0.998,1.055)
		Prop. Mediated		0.484*	(0.171,2.708)	0.561	(-1.665,4.083)
Fall (N=789)	30-50	ACME		1.004	(0.988,1.02)	1.003	(0.99,1.016)
		ADE		1.022	(0.949,1.103)	1.019	(0.964,1.083)
		TE		1.026	(0.953,1.11)	1.021	(0.963,1.089)
		Prop. Mediated		0.092	(-2.352,1.975)	0.073	(-1.624,2.067)
	<30	ACME		1.02	(0.998,1.045)	1.015	(0.998,1.038)
		ADE		1.101	(0.992,1.223)	1.051	(0.971,1.152)
		TE		1.123*	(1.006,1.248)	1.067	(0.982,1.168)
		Prop. Mediated		0.167	(-0.051,0.91)	0.210	(-1.168,2.078)
Dpression (N=273)	30-50	ACME		1.002	(0.973,1.034)	1.058	(0.987,1.15)
		ADE		1.052	(0.893,1.216)	1.011	(0.892,1.144)
		TE		1.054	(0.9,1.223)	1.070	(0.919,1.238)
		Prop. Mediated		0.031	(-1.314,1.701)	0.612	(-4.103,5.374)
	<30	ACME		1.016	(0.985,1.061)	1.048	(0.978,1.141)
		ADE		1.051	(0.872,1.261)	1.022	(0.879,1.235)
		TE		1.068	(0.882,1.289)	1.070	(0.896,1.287)
		Prop. Mediated		0.082	(-1.81,1.511)	0.439	(-4.294,7.901)
Loneliness (N=264)	30-50	ACME		1.005	(0.975,1.039)	1.035	(0.987,1.098)
		ADE		1.054	(0.897,1.227)	1.068	(0.931,1.218)
		TE		1.059	(0.903,1.238)	1.105	(0.953,1.269)
		Prop. Mediated		0.048	(-1.783,1.502)	0.289	(-1.161,2.053)
	<30	ACME		1.039	(0.999,1.098)	1.080**	(1.019,1.167)
		ADE		0.980	(0.826,1.171)	1.012	(0.885,1.169)
		TE		1.017	(0.854,1.217)	1.093	(0.947,1.253)
		Prop. Mediated		0.163	(-6.629,7.065)	0.720	(-7.505,6.738)
Living alone (N=760)	30-50	ACME		0.999	(0.987,1.011)	1.002	(0.987,1.018)
		ADE		1.056	(0.976,1.142)	1.089**	(1.023,1.161)
		TE		1.056	(0.976,1.143)	1.092**	(1.026,1.169)
		Prop. Mediated		-0.002	(-0.645,0.613)	0.025	(-0.217,0.269)
	<30	ACME		1.020**	(1.004,1.041)	1.023**	(1.004,1.045)
		ADE		0.993	(0.911,1.089)	1.075*	(1.008,1.149)
		TE		1.013	(0.929,1.114)	1.099**	(1.028,1.18)
		Prop. Mediated		0.261	(-5.813,5.013)	0.239**	(0.054,0.706)
Hypertension (N=1030)	30-50	ACME		1.001	(0.987,1.014)	1.003	(0.996,1.011)
		ADE		1.082*	(1.010,1.160)	1.068**	(1.017,1.122)
		TE		1.083*	(1.011,1.163)	1.071**	(1.021,1.127)
		Prop. Mediated		0.012	(-0.299,0.245)	0.040	(-0.078,0.204)
	<30	ACME		1.035**	(1.014,1.06)	1.015**	(1.004,1.028)

		ADE	1.038	(0.960,1.125)	1.072**	(1.018,1.131)
		TE	1.074	(0.989,1.174)	1.088**	(1.033,1.150)
		Prop. Mediated	0.459	(-1.303,2.799)	0.168**	(0.055,0.482)
**Diabetes (N=227)**	30-50	ACME	0.993	(0.960,1.023)	-	-
		ADE	1.018	(0.884,1.176)	-	-
		TE	1.011	(0.878,1.170)	-	-
		Prop. Mediated	0.014	(-2.415,3.868)	-	-
	<30	ACME	1.031	(0.997,1.082)	-	-
		ADE	0.862	(0.745,1.006)	-	-
		TE	0.889	(0.764,1.037)	-	-
		Prop. Mediated	-0.194	(-2.741,2.486)	-	-
**Arthritis (N=1100)**	30-50	ACME	1.009	(0.997,1.023)	1.015*	(1.002,1.032)
		ADE	1.088*	(1.013,1.167)	1.035	(0.975,1.101)
		TE	1.099*	(1.024,1.179)	1.051	(0.989,1.120)
		Prop. Mediated	0.096	(-0.055,0.398)	0.277	(-1.495,1.999)
	<30	ACME	1.021*	(1.005,1.040)	1.021**	(1.005,1.041)
		ADE	1.069	(0.982,1.165)	1.004	(0.942,1.083)
		TE	1.091*	(1.001,1.192)	1.025	(0.961,1.109)
		Prop. Mediated	0.227	(-0.004,1.590)	0.406	(-6.511,6.415)
**Obese (N=878)**	30-50	ACME	1.006	(0.991,1.021)	1.010	(0.996,1.026)
		ADE	1.103*	(1.025,1.180)	1.018	(0.957,1.088)
		TE	1.110**	(1.029,1.188)	1.028	(0.965,1.101)
		Prop. Mediated	0.058	(-0.124,0.26)	0.213	(-3.240,2.705)
	<30	ACME	1.028**	(1.009,1.053)	1.023**	(1.004,1.045)
		ADE	1.049	(0.963,1.152)	1.009	(0.942,1.084)
		TE	1.078	(0.987,1.186)	1.032	(0.961,1.115)
		Prop. Mediated	0.337	(-1.664,2.681)	0.428	(-5.286,7.853)
**Smoking (N=316)**	30-50	ACME	1.019	(0.997,1.052)	1.009	(0.980,1.042)
		ADE	1.022	(0.920,1.142)	1.034	(0.959,1.113)
		TE	1.042	(0.935,1.169)	1.043	(0.966,1.132)
		Prop. Mediated	0.198	(-4.687,3.691)	0.176	(-1.993,2.388)
	<30	ACME	1.063**	(1.020,1.122)	1.064**	(1.021,1.119)
		ADE	1.137	(0.987,1.313)	1.051	(0.978,1.144)
		TE	1.209**	(1.050,1.385)	1.118*	(1.024,1.237)
		Prop. Mediated	0.315**	(0.088,1.174)	0.567*	(0.206,1.546)
**Social isolation (N=413)**	30-50	ACME	1.016*	(1.001,1.042)	1.010	(0.997,1.029)
		ADE	1.042	(0.943,1.156)	0.981	(0.895,1.084)
		TE	1.058	(0.956,1.174)	0.990	(0.903,1.092)
		Prop. Mediated	0.194	(-2.601,3.049)	-0.059	(-3.392,2.156)
	<30	ACME	1.012	(0.992,1.042)	1.009	(0.996,1.029)
		ADE	0.997	(0.886,1.134)	0.971	(0.892,1.057)
		TE	1.009	(0.897,1.147)	0.980	(0.901,1.068)
		Prop. Mediated	0.042	(-2.807,2.490)	-0.079	(-2.340,2.330)

Note: Abbreviations: ACME, the average causal mediation effect; ADE, the average direct effect; TE, total effect; Prop. Mediated, the mediating propotion; CI, confidence interval. In all models, ACME, ADE and TE were translated into the corresponding odds ratio (OR) values. *P < 0.05; **P < 0.01

Supporting Information Table 6. Association between different 25(OH)D concentration and frailty trajectories by gender

Sex	25(OH)D level (ref: ≥50 nmol/L)	Model 1					Model 2				
		Non-frail	Pre-frail to frail		Frail to severely frail		Non-frail	Pre-frail to frail		Frail to severely frail	
			OR (95%CI)	p-value	OR (95%CI)	p-value		OR (95%CI)	p-value	OR (95%CI)	p-value
**Male (N=1334)**	30-50		1.32 (0.92,1.89)	0.139	1.38 (0.60,3.18)	0.451		1.17 (0.81,1.69)	0.415	1.26 (0.53,3.01)	0.603
	<30		1.82* (1.14,2.90)	0.012	2.46 (1.00,6.05)	0.050		1.49 (0.92,2.41)	0.103	1.73 (0.67,4.45)	0.258
**Female (N=1663)**	30-50		1.18 (0.88,1.57)	0.273	1.29 (0.77,2.15)	0.333		1.13 (0.84,1.52)	0.422	1.26 (0.74,2.15)	0.388
	<30		1.38 (0.98,1.95)	0.065	2.25** (1.31,3.86)	0.003		1.22 (0.86,1.73)	0.268	1.82* (1.03,3.19)	0.038

Note: Abbreviations: OR, odds ratio; CI, confidence interval. Model 1 is adjusted for education, marital status, employment, wealth, smoking, alcohol intake, BMI, VD supplements use and season. Model 2 is further adjusted for physical activity at wave 6. *P < 0.05; **P < 0.01
